# Pre-Pregnancy Body Mass Index in Relation to Infant Birth Weight and Offspring Overweight/Obesity: A Systematic Review and Meta-Analysis

**DOI:** 10.1371/journal.pone.0061627

**Published:** 2013-04-16

**Authors:** Zhangbin Yu, Shuping Han, Jingai Zhu, Xiaofan Sun, Chenbo Ji, Xirong Guo

**Affiliations:** State Key Laboratory of Reproductive Medicine, Department of Pediatrics, Nanjing Maternity and Child Health Care Hospital, Nanjing Medical University, Nanjing, China; Tehran University of Medical Sciences, Iran (Islamic Republic of)

## Abstract

**Background:**

Overweight/obesity in women of childbearing age is a serious public-health problem. In China, the incidence of maternal overweight/obesity has been increasing. However, there is not a meta-analysis to determine if pre-pregnancy body mass index (BMI) is related to infant birth weight (BW) and offspring overweight/obesity.

**Methods:**

Three electronic bibliographic databases (MEDLINE, EMBASE and CINAHL) were searched systematically from January 1970 to November 2012. The dichotomous data on pre-pregnancy overweight/obesity and BW or offspring overweight/obesity were extracted. Summary statistics (odds ratios, ORs) were used by Review Manager, version 5.1.7.

**Results:**

After screening 665 citations from three electronic databases, we included 45 studies (most of high or medium quality). Compared with normal-weight mothers, pre-pregnancy underweight increased the risk of small for gestational age (SGA) (odds ratios [OR], 1.81; 95% confidence interval [CI], 1.76–1.87); low BW (OR, 1.47; 95% CI, 1.27–1.71). Pre-pregnancy overweight/obesity increased the risk of being large for gestational age (LGA) (OR, 1.53; 95% CI, 1.44–1.63; and OR, 2.08; 95% CI; 1.95–2.23), high BW (OR, 1.53; 95% CI, 1.44–1.63; and OR, 2.00; 95% CI; 1.84–2.18), macrosomia (OR, 1.67; 95% CI, 1.42–1.97; and OR, 3.23; 95% CI, 2.39–4.37), and subsequent offspring overweight/obesity (OR, 1.95; 95% CI, 1.77–2.13; and OR, 3.06; 95% CI, 2.68–3.49), respectively. Sensitivity analyses revealed that sample size, study method, quality grade of study, source of pre-pregnancy BMI or BW had a strong impact on the association between pre-pregnancy obesity and LGA. No significant evidence of publication bias was observed.

**Conclusions:**

Pre-pregnancy underweight increases the risk of SGA and LBW; pre-pregnancy overweight/obesity increases the risk of LGA, HBW, macrosomia, and subsequent offspring overweight/obesity. A potential effect modification by maternal age, ethnicity, gestational weight gain, as well as the role of gestational diseases should be addressed in future studies.

## Introduction

Overweight/obesity in women of childbearing age is a serious public-health problem, especially in “developing” countries. In China, from 1992 to 2010, the prevalence of overweight or obesity in women aged 18–44 years increased from 16.8% to 26.4%, and from 3.1% to 9.0%, respectively [1]–[2]. Worryingly, these estimates of prevalence are higher in “developed” nations. In the UK, the prevalence of maternal obesity has more than doubled from 7.6% to 15.6% from 1989 to 2007, respectively [3]. In women aged 20–39 years residing in North America, the prevalence of obesity increased from 13.0% to 22.0% from 1993 to 2003 [4]. In 2008, data from the Pregnancy Nutrition Surveillance System of USA showed that the prevalence of pre-pregnancy obesity increased to 28.5% [5].

The impact of pre-pregnancy body mass index (BMI) on pregnant and neonatal outcomes, as well as subsequent disease risk in the offspring, has attracted widespread attention. Pre-pregnancy underweight has been shown to increase the risk of preterm birth and low birth weight (BW) [6], as well as to increase the risk of subsequent obesity and hypertension in the offspring [7]. Pre-pregnancy overweight/obesity is a risk factor for diabetes mellitus (DM), hypertension, and preeclampsia in pregnancy [8]–[10]. However, it also increases the risk of caesarean and instrumental deliveries, hemorrhage, infection and maternal mortality during labor [11]–[14]. Pre-pregnancy overweight/obesity has been shown to increase the risk of adverse neonatal outcome (e.g., preterm delivery, low/high BW, congenital anomalies, neonatal asphyxia, neonatal death, hypoglycemia, and hyperbilirubinemia), increased requirement for neonatal intensive care, and a longer duration of hospital stay [15]–[18]. Maternal overweight/obesity carries an increased risk of subsequent disease risk in the offspring. This can include impaired neurodevelopmental outcome (cognitive problems, attention deficit hyperactivity disorder, and psychotic disorders), asthma, schizophrenia, insulin resistance, DM, hypertension, coronary heart disease, stroke, and even death [19]–[23].

In recent years, evidence has accumulated and supported the notion that the intrauterine environment can “program” or affect pregnancy and neonatal outcomes, as well as subsequent long-term health and development in the offspring; this is referred to as the “fetal programming” or “fetal origins hypothesis” [24]. BW is frequently used as an indicator of the conditions experienced *in utero* [25]. The association between BW and subsequent obesity in the also has been confirmed [26]. Pre-pregnancy BMI has an impact on BW [27]. Therefore, we suspect that BW may be a key feature explaining the association between pre-pregnancy BMI and subsequent obesity in the offspring.

Hence, maternal BMI during pre-pregnancy can affect overweight/obesity in the offspring. In addition, it may be a modifiable risk factor for childhood overweight/obesity if the BW is optimized. Therefore, this area is of particular worth as a study area. Reviews on this topic have been limited by the use of qualitative methodology analyzing a limited number of studies [22], [28]. Therefore, we carried out a systematic review of extant studies to determine if pre-pregnancy BMI is related to the BW of infants and overweight/obesity in the offspring.

## Methods

This systematic review and meta-analysis was conducted according to the guidelines for the Meta-analysis of Observational Studies in Epidemiology (MOOSE) [29].

### Study selection

Observational studies (cohort, case-control, and cross-sectional) were included irrespective of publication status, sample size, follow-up duration, or language. Studies defined pre-pregnancy BMI categories according to different standards. The first was according to the recommendation of Abrams and Parker [30]: underweight (BMI<20 kg m^−2^), normal weight (20–24.9 kg m^−2^), overweight (25–29.9 kg m^−2^), and obese (≥30 kg m^−2^). The second was according to the World Health Organization (WHO) classification [31]: underweight (<18.5 kg m^−2^), normal weight (18.5–24.9 kg m^−2^), overweight (25–29.9 kg m^−2^), and obese (≥30 kg m^−2^). The third was according to recommendations set by the Institute of Medicine (IOM) [32]: underweight (<19.8 kg m^−2^), normal weight (19.8–26.0 kg m^−2^), overweight (26.1–29.0 kg m^−2^), and obese (>29.0 kg m^−2^). The fourth was according to the BMI classification for Chinese adults proposed by the Working Group on Obesity in China (WGOC) in 2001 [33]: underweight (<18.5 kg m^−2^), normal weight (18.5–23.9 kg m^−2^), overweight (24.0–27.9 kg m^−2^), and obese (≥28.0 kg m^−2^). The fifth was according to the Asia-Pacific standard (APS) [34]: underweight (<18.5 kg m^−2^), normal weight (18.5–22.9 kg m^−2^), overweight (23.0–24.9 kg m^−2^), and obese (≥25.0 kg m^−2^). Studies have been carried out that define BW categories [35]. That is, large- for-gestational-age (LGA) and small-for-gestational-age (SGA) births were defined if BWs were above the 90th percentile and below the 10th percentile, respectively, using gestational age- and sex-specific reference curves. High birth weight (HBW) and low birth weight (LBW) births were assessed by BW irrespective of gestational age, and corresponded to >4,000 g and <2,500 g, respectively. Macrosomia was defined as BW ≥4,500 g. Studies that defined offspring overweight/obesity categories according to the BMI were included. The Centers for Disease Control and Prevention (CDC) [29] and International Obesity Task Force (IOTF) [21] have separately published BMI reference standards for children and adolescents; overweight was defined as a BMI more than the 85th percentile but less than the 95th percentile according to sex and age, whereas obesity was defined as a BMI above the 95th percentile. Studies that classified overweight/obesity in offspring according to the deviation from the ideal weight-for-height recommended by the WHO were also included. In these studies, the ratio of weight (W) to ideal weight (IW) was calculated; overweight was defined as W/IW>1.1, and obesity as W/IW>1.2 [36]. Pre-pregnancy BMI, infant BW or offspring overweight/obesity were recorded from self-reported statements, medical records or obtained by interview or questionnaire.

### Data sources and search strategies

The search strategy was developed with the assistance of a librarian (Q Tang) experienced in systematic reviews based at Southeast University (Nanjing, China), and was adapted for each database searched. The search term was “pregnancy”, “pre-pregnancy”, “body mass index”, “obesity”, “overweight”, “birth weight”, “childhood”, “infant”, “adolescence”. (please see Appendix S1).

Three electronic bibliographic databases (MEDLINE, EMBASE and CINAHL) were searched systematically from January 1970 to November 2012. There were no restrictions regarding language or country. Searching of gray literature and hand-searching was not performed. If data in the original publication were not sufficiently detailed, the authors were contacted for additional information. The reference list of included studies should be searched for addition eligible studies.

### Screening and data-extraction form

All citations identified by electronic databases were organized, duplicates deleted, and each citation assigned a unique identification number. Initially, two investigators (ZB Yu and JG Zhu) independently screened the results of the electronic searches to select potentially relevant citations based on titles and abstracts. Discrepancies were resolved through consensus. If the citation was relevant or if the title/abstract was not sufficient for deciding on inclusion/exclusion, full texts were retrieved and evaluated. All articles selected at first screening were read and abstracted independently by the two reviewers (ZB Yu and XF Sun). Differences between the two reviewers were resolved by consensus or referred to a third reviewer (CB Ji) if necessary. Information extracted from each article included: publication year, country, study design, study period, source of study population, source of pre-pregnancy BMI or BW, diagnostic criteria for pre-pregnancy or offspring overweight/obesity, study size, and confounding factors. An independent reviewer (XF Sun) confirmed all data entries. Raw data for the exposed, non-exposed, outcome, and non-outcome groups were obtained if possible. Otherwise, odds ratios (ORs) were recorded, with preference given to crude ORs or adjusted ORs.

### Quality assessment

To assess the quality of included studies, we created a specific Quality Assessment Scale (Appendix S2) based on the criteria proposed by Strengthening the Reporting of Observational Studies in Epidemiology and Tooth *et al.* for the assessment of observational studies [37]. Briefly, we assessed the quality of all included studies in accordance with the following items: type of study, loss of follow-up, sample size, participant selection, comparability of groups, statistical method, and diagnostic criteria for pre-pregnancy overweight/obesity, measurement of BW/offspring overweight or obesity. According to the score achieved (from 0 to 18), studies were classified as being of high (>14), medium (11–14) or low (<11) quality.

### Statistical analyses

If we could not obtain sufficient dichotomous data on pre-pregnancy overweight/obesity and BW or offspring overweight/obesity from these studies (which presented crude ORs or adjusted ORs on the association), we included these studies in the systematic review. Studies that could construct separate 2×2 tables to calculate the ORs and 95% confidence intervals (CIs) were included in the meta-analysis. The chi-squared test was used to test for heterogeneity across studies. A random effects model was used to account for possible heterogeneity between studies, which defaults to the fixed effects model approach in the absence of heterogeneity [38]. *P*<0.01 was considered significant. Statistical analyses were conducted using Review Manager, ver5.1.7 (Nordic Cochrane Center, Copenhagen, Denmark). We undertook subgroup analyses according to the different pre-pregnancy categories of the BMI, which compared pre-pregnancy underweight, overweight, obesity and pre-pregnancy normal weight. We also undertook subgroup analyses according to the different categories of BW: SGA, LGA, LBW, HBW, and macrosomia. Sensitivity analyses were carried out to determine differences in statistical method, study design, sample size, quality grade of the study, and diagnostic criteria for pre-pregnancy overweight/obesity. Publication bias was assessed by inspection of the funnel plot and formal testing for asymmetry of the funnel plot using Egger's test [39]. These calculations were carried out using Stata/SE, ver9 (Stata, College Station, TX, USA).

## Results

### Description of studies

**Figure 1:**
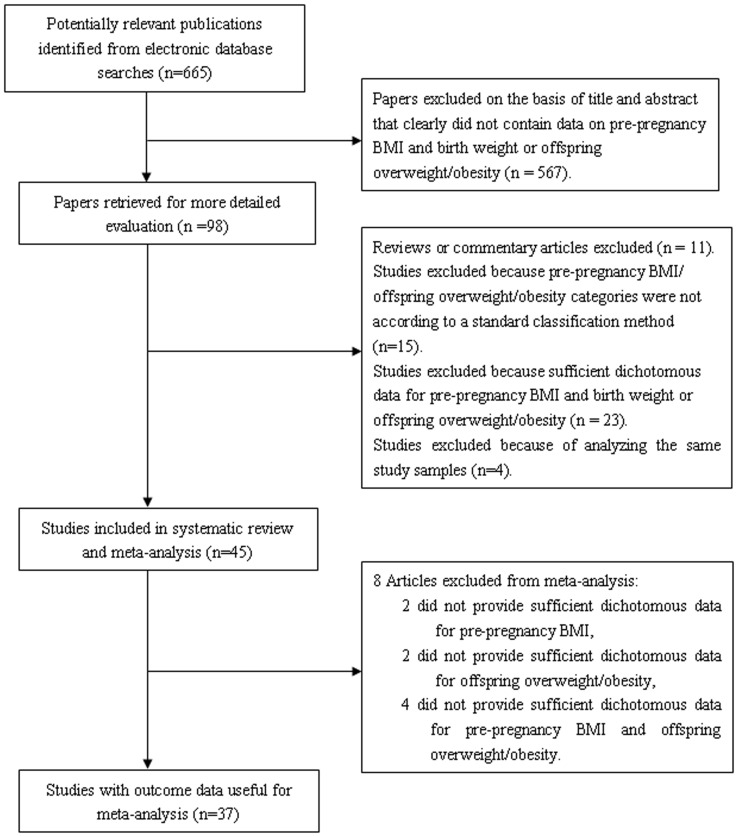
Screening and selection process for articles.

A search of three electronic databases identified 665 articles, 620 of which were excluded based on the reasons listed in Figure 1. Forty-five articles were included in the systematic review and meta-analysis [13], [16], [40]–[82]: 3 case–control [44], [48], [57], 4 cross-sectional [62], [63], [73], [80], and 38 cohort [13], [16], [40]–[43], [45]–[47], [49]–[56], [58]–[61], [64]–[72], [74]–[79], [81], [82] studies. In one of these studies, the impact of pre-pregnancy BMI on BW and overweight/obesity in offspring was assessed [54]. In the remaining 44 articles, 33 articles [13], [16], [40]–[53], [55]–[71] investigated the association between pre-pregnancy BMI and BW (the descriptive information for each included study is presented in Table S1). Eleven articles [72]–[82] analyzed the impact of pre-pregnancy BMI on offspring overweight/obesity (descriptive information for each included study is presented in Table S2).

In 45 studies of pre-pregnancy categories of the BMI, 10 studies were according to the recommendation of Abrams and Parker [40], [41], [43], [45], [49], [55], [58], [62], [64], [80], 24 studies were according to the classification set by the WHO [16], [42], [47], [48], [51]–[54], [56], [57], [65], [69]–[79], [81], [82], 8 studies were according to the IOM recommendations [44], [46], [50], [60], [61], [66]–[68], 2 studies were according to the classification proposed by the WGOC [13], [59] and 1 study was according to the APS [63]. According to the BW categories, SGA were investigated in 16 studies [13], [16], [41], [47], [48], [51], [53]–[57], [61], [66], [69]–[71], LGA in 21 studies [13], [16], [40], [42], [47]–[51], [53]–[57], [59]–[61], [66], [69]–[71], LBW in 10 studies [41], [43], [45], [46], [48], [61], [63], [65], [68], [71], HBW in 12 studies [41]–[43], [45], [46], [49], [58], [61], [63], [65], [67], [71] and macrosomia in 10 studies [44], [48]–[50], [52], [53], [57], [58], [62], [64]. According to the categories of overweight/obesity in offspring, 6 studies were according to CDC recommendations [54], [74]–[77], [82], 5 studies were according to IOTF recommendations [72], [73], [78], [80], [81] and 1 study was according to the classification set by the WHO (W/IW) [79]. The quality of each study is summarized in Appendices S3. Six studies received scores of ≥15 and were considered to be of high methodological quality [40], [43], [50], [53], [78], [80]. Nineteen studies received scores between 11 and 14, and were considered to be of medium methodological quality [16], [41], [45], [47], [49], [51], [54], [55], [59]–[61], [66], [67], [69], [70], [72], [74], [75], [81]. The remaining 20 studies received scores of ≤10 and were considered to be of low methodological quality [13], [42], [44], [46], [48], [52], [56]–[58], [62]–[65], [68], [71], [73], [76], [77], [79], [82]. The PRISMA statement see checklist S1.

### Effect of pre-pregnancy BMI on infant BW

**Figure 2:**
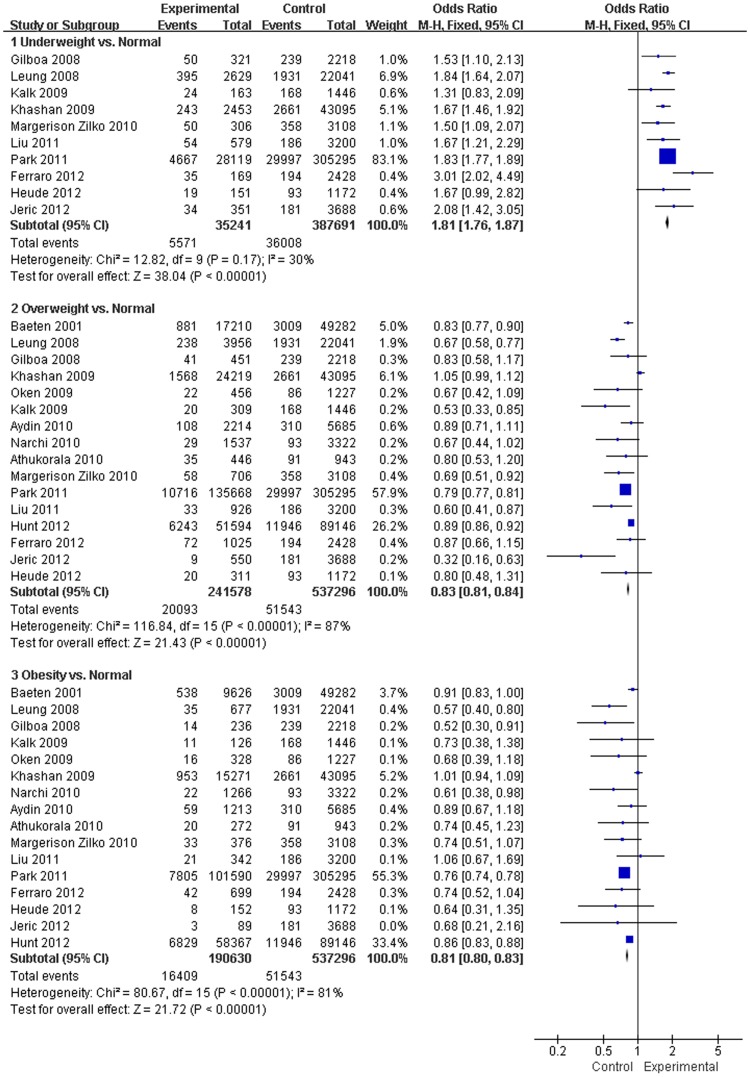
Forest plot of the association between pre-pregnancy BMI and being SGA.

Thirty-four articles [13], [16], [40]–[71] investigated the association between pre-pregnancy BMI and infant BW. Sixteen studies assessed the association between pre-pregnancy BMI and SGA [13], [16], [41], [47], [48], [51], [53]–[57], [61], [66], [69]–[71]. In comparison with a mother with a normal BMI, the results from this analysis revealed that pre-pregnancy underweight increased the risk of SGA (OR, 1.81; 95% CI, 1.76–1.87; *P*<0.001) (Figure 2). In contrast, pre-pregnancy overweight or obesity decreased the risk of LBW in the meta-analysis (OR, 0.83; 95% CI, 0.81–0.84; and OR, 0.81; 95% CI, 0.80–0.83; *P*<0.001) (Figure 2).

**Figure 3:**
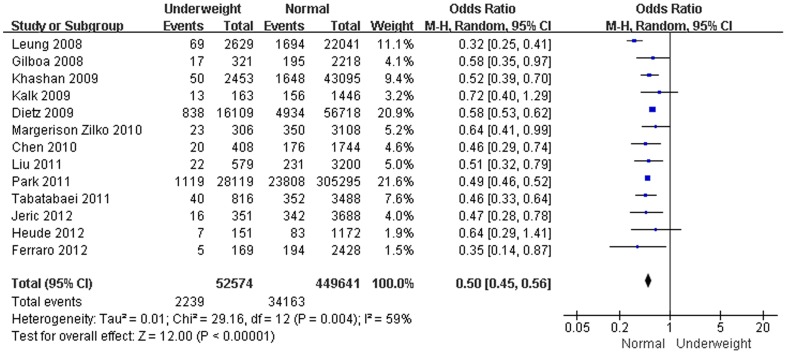
Forest plot of the association between pre-pregnancy underweight and being LGA.

**Figure 4:**
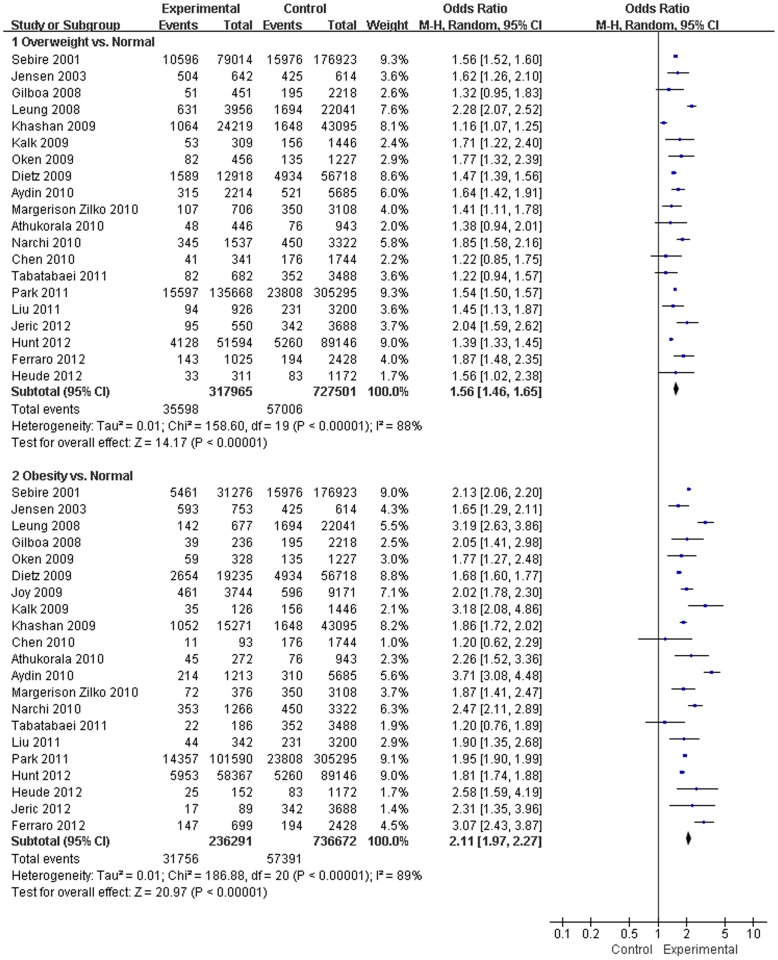
Forest plot of the association between pre-pregnancy overweight or obesity and being LGA.

Twenty-one studies assessed the association between pre-pregnancy BMI and LGA [13], [16], [40], [42], [47]–[51], [53]–[57], [59]–[61], [66], [69]–[71]. In comparison with a mother with a normal BMI, the results from this analysis revealed that pre-pregnancy underweight decreased the risk of LGA (OR, 0.51; 95% CI, 0.46–0.56; *P*<0.001) (Figure 3). In contrast, pre-pregnancy overweight or obesity increased the risk of LGA in the meta-analysis (OR, 1.53; 95% CI, 1.44–1.63; and OR, 2.08; 95% CI, 1.95–2.23; *P*<0.001) (Figure 4).

Ten studies assessed the association between pre-pregnancy BMI and LBW [41], [43], [45], [46], [48], [61], [63], [65], [68], [71]. In comparison with a mother with a normal BMI, the results from this analysis revealed that pre-pregnancy underweight increased the risk of LBW (OR, 1.47; 95% CI, 1.27–1.71; *P*<0.001) (Figure S1). In contrast, no significant association was revealed between pre-pregnancy overweight or obesity and LBW in the meta-analysis (OR, 0.88; 95% CI, 0.77–1.00; and OR, 1.09; 95% CI, 0.87–1.37; *P*>0.010) (Figure S1).

Twelve studies assessed the association between pre-pregnancy BMI and HBW [41]–[43], [45], [46], [49], [58], [61], [63], [65], [67], [71]. We pooled the data from these studies and revealed a negative association between pre-pregnancy underweight and HBW (OR, 0.51; 95% CI, 0.43–0.61; *P*<0.001) (Figure S2). In contrast, pre-pregnancy overweight or obesity was associated with an increased risk of HBW in comparison with subjects with a normal BMI in the meta-analysis (OR, 1.53; 95% CI, 1.44–1.63; and OR, 2.00; 95% CI, 1.84–2.18; *P*<0.001) (Figure S2).

Ten studies assessed the association between pre-pregnancy BMI and macrosomia [44], [48]–[50], [52], [53], [57], [58], [62], [64]. We pooled the data from these studies and revealed a negative association between pre-pregnancy underweight and macrosomia (OR, 0.51; 95% CI, 0.42–0.61; *P*<0.001) (Figure S3). In contrast, pre-pregnancy overweight or obesity was associated with an increased risk of macrosomia in comparison with subjects with a normal BMI in the meta-analysis (OR, 1.67; 95% CI, 1.42–1.97; and OR, 3.23; 95% CI, 2.39–4.37; *P*<0.001) (Figure S3).

### Effect of pre-pregnancy BMI on overweight/obesity in offspring

Twelve reports evaluated the association between pre-pregnancy BMI and overweight/obesity in offspring [54], [72]–[82]. Only 4 studies [54], [73], [79], [81] provided sufficient dichotomous data for pre-pregnancy BMI and offspring overweight/obesity, and were included in the meta-analysis. Results from this analysis revealed a negative association between pre-pregnancy underweight and offspring overweight/obesity (OR, 0.46; 95% CI, 0.37–0.56; *P*<0.001) (Figure S4). In contrast, pre-pregnancy overweight or obesity was associated with an increased risk of offspring overweight/obesity in comparison with subjects with a normal BMI in the meta-analysis (OR, 1.95; 95% CI, 1.77–2.13; and OR, 3.06; 95% CI, 2.68–3.49; *P*<0.001) (Figure S4).

The remaining 8 studies reported the outcomes using insufficient dichotomous data and could not be pooled by the meta-analysis. The results of these studies were non-conforming. Whitaker *et al.* [72] reported a retrospective cohort study in 8,494 children from low-income families who were enrolled in the Special Supplemental Nutrition Program for Women, Infants, and Children in Ohio, USA; a follow-up survey was conducted at ages 2, 3 and 4 years. That study found that pre-pregnancy underweight was associated with a decreased prevalence of childhood obesity; pre-pregnancy overweight or obesity was associated with an increased risk of childhood obesity at ages 2, 3 and 4 years.

Li *et al.* [74] and Salsberry *et al.* [75] analyzed the 1996 National Longitudinal Survey of Youth, Child and Young Adult data in the USA. After adjusting for potential confounders, Li *et al.* [74] revealed that children at 2–14 years of age whose mothers were obese before pregnancy were also at a greater risk of becoming obese (OR, 4.1; 95% CI, 2.6–6.4; *P*<0.001) than children whose mothers had a normal BMI. Salsberry *et al.* [75] also found the same results at follow-up of 2–3, 4–5, 6–7 years of age. The results from this analysis revealed a negative association between pre-pregnancy underweight and overweight/obesity in offspring (OR, 0.46; 95% CI, 0.37–0.56; *P*<0.001) (Figure 3). In contrast to the results of the meta-analysis described above, Salsberry *et al.* [75] showed that pre-pregnancy underweight was not significantly associated with an increased risk of offspring obesity. Dubois *et al.* [76] analyzed the data from the Quebec Longitudinal Study of Child Development 1998–2002, which also showed that pre-pregnancy underweight was not significantly associated with increased risk of offspring obesity at a follow-up of 4.5 years of age (OR, 0.7; 95% CI, 0.3–1.9; *P*>0.010).

Hawkins *et al.* [78] analyzed a prospective, nationally representative millennium cohort study in which 13,188 singleton children were enrolled. They showed that pre-pregnancy overweight was significantly associated with an increased risk of offspring overweight at a follow-up of 3 years of age (OR, 1.83; 95% CI, 1.66–2.02; *P*<0.001). Maddah *et al.* [80] investigated 6,635 children attending elementary schools in Rasht, Iran, by gathering data on pre-pregnancy BMI using a self-administrated questionnaire. After adjusting for potential confounders, pre-pregnancy overweight/obesity was shown to be associated with an increased risk of childhood overweight/obesity at ages 6–11 years (OR, 1.6; 95% CI, 1.1–2.3; *P*<0.001).

Two studies chose mothers with underweight and normal weight as the control, not mothers with normal weight. Hernandez-Valero *et al.* [77] undertook a population-based Mexican–American cohort study and found that pre-pregnancy obesity was significantly associated with an increased risk of offspring obesity at a follow-up of 5–18 years of age (OR, 2.14; 95% CI, 1.12–4.08; *P*>0.001). Janjua *et al.* [82] analyzed the data from a longitudinal study of pregnancy outcomes and childhood psychomotor development. They also revealed that pre-pregnancy obesity was significantly associated with an increased risk of offspring obesity at a follow-up of 5 years of age (OR, 2.92; 95% CI, 1.73–4.91; *P*>0.001).

### Analyses of heterogeneity and publication bias

Heterogeneity (*I^2^*>50%) was high for the pooled ORs of the studies in the meta-analysis. The *x* ^2^-test for heterogeneity was significant for the 21 studies investigating the association between pre-pregnancy obesity and LGA (*x* ^2^ = 186.88, *P*<0.001), and this was taken into account by analyzing the data using a random model. Sensitivity analyses were carried out (Table 1), and subgroups were divided based on the differences in statistical method, study design, study method, sample size, quality grade of study, source of pre-pregnancy BMI, pre-pregnancy BMI categories, distribution of pre-pregnancy BMI, source of BW, and the geographic location of the study.

**Table 1:** Sensitivity analyses of the relationship between pre-pregnancy obesity and being LGA.

*Subgroup*	*Number of studies (n)*	*Pooled ORs (95% CI)*	*P*	*I^2^*	*P*
Statistical method					
Fixed effect	21	1.95 (1.92, 1.98)	<0.001	89%	<0.001
Random effect	21	2.11 (1.97, 2.27)	<0.001	89%	<0.001
Study design					
Prospective	6	2.09 (1.83, 2.40)	<0.001	94%	<0.001
Retrospective	15	1.93 (1.89, 1.97)	<0.001	86%	<0.001
Study method					
Case–control	2	2.15 (1.64, 2.82)	<0.001	0%	0.730
Cohort	19	2.11 (1.96, 2.27)	<0.001	90%	<0.001
Sample size					
≥5,000	11	1.94 (1.91, 1.97)	<0.001	94%	<0.001
<5,000, ≥2,000	8	2.11 (1.70, 2.63)	<0.001	69%	0.002
<2,000	2	2.38 (1.75, 3.23)	<0.001	0%	0.680
Quality grade of study					
High	2	1.89 (1.50, 2.39)	<0.001	98%	<0.001
Medium	6	2.15 (1.92, 2.40)	<0.001	39%	0.150
Low	13	2.19 (1.99, 2.42)	<0.001	89%	<0.001
Source of pre-pregnancy BMI					
Recorded from medical records	12	2.09 (1.93, 2.26)	<0.001	92%	<0.001
Self-reported	5	1.70 (1.41, 2.05)	<0.001	18%	0.300
Questionnaire	2	3.14 (2.71, 3.64)	<0.001	0%	0.800
Measured by research assistants	2	2.00 (1.53, 2.62)	<0.001	42%	0190
Pre-pregnancy categories of BMI					
Abrams and Parker	3	2.48 (1.91, 3.23)	<0.001	94%	<0.001
WHO	13	2.23 (1.91, 2.59)	<0.001	78%	<0.001
IOM	4	1.79 (1.64, 1.95)	<0.001	92%	<0.001
WGOC	1	1.90 (1.35, 2.68)	<0.001	-	-
Distribution of pre-pregnancy BMI					
Four groups	13	2.19 (1.91, 2.52)	<0.001	93%	<0.001
Three groups	7	2.08 (1.88, 2.31)	<0.001	86%	<0.001
Two groups	1	2.02 (1.78, 2.30)	<0.001	-	-
Source of BW					
Recorded from medical records	8	1.86 (1.63, 2.14)	<0.001	66%	0.005
Not reported	7	2.28 (1.95, 2.66)	<0.001	88%	<0.001
Questionnaire	1	3.19 (2.63, 3.86)	<0.001	-	-
Measured by research assistants	2	2.93 (2.37, 3.63)	<0.001	0%	0.340
Reported by mothers	1	1.87 (1.41, 2.47)	<0.001	-	-
Date from birth certificate	2	1.88 (1.75, 2.02)	<0.001	90%	<0.001
Geographic location of study					
Asia	4	1.79 (1.06, 3.01)	<0.001	87%	<0.001
North America	8	1.92 (1.78, 2.07)	<0.001	86%	<0.001
European	8	2.34 (2.02, 2.72)	<0.001	88%	<0.001
Oceania	1	2.26 (1.52, 3.36)	<0.001	-	-

BMI, body mass index; BW, birth weight; WHO, World Health Organization; IOM, Institute of Medicine; WGOC, Working Group on Obesity in China; APS, Asia-Pacific standard; LGA, large for gestational age.

The results showed that the differences in sample size, study method, quality grade of study, and source of pre-pregnancy BMI or infant BW made a strong impact on the association between pre-pregnancy obesity and LGA. Inspection of funnel plots did not reveal an obvious effect of publication bias, and Egger's test for publication bias was not significant (*P* = 0.813) for studies investigating the association between pre-pregnancy obesity and LGA (Appendix S4).

## Discussion

The present comprehensive systematic review and meta-analysis indicated: that pre-pregnancy underweight increased the risk of SGA and LBW; that pre-pregnancy overweight or obesity increased the risk of LGA, HBW, macrosomia; subsequent offspring overweight/obesity in comparison with mothers with a normal BMI. The present study suggests inconsistency regarding the association between pre-pregnancy underweight and offspring overweight/obesity. Further prospective studies are needed to examine whether a causative relationship between pre-pregnancy underweight and offspring overweight/obesity exists.

The systematic review provided here was developed by a robust search strategy. Furthermore, we strove to obtain information following the MOOSE recommendations. The prevalence of pre-pregnancy overweight/obesity is increasing in many parts of the world. Acceptance of the problem and subsequent epidemiological studies have begun in recent years, as reflected by the fact that 66.7% of the studies identified for this review were conducted in between 2009 and 2012.

Sources of bias in any meta-analysis are the selection and heterogeneity of the included studies. In this regard, a specific limitation of our systematic review and meta-analysis is related to the difficulty of combining studies that used different methods to assess and classify the exposure (pre-pregnancy BMI) and outcome (infant BW and offspring overweight/obesity) of the participants. This is related directly to the lack of consensus about the categorization of pre-pregnancy BMI and offspring overweight/obesity.

We undertook subgroup analyses to evaluate other sources of bias in the review (Table 1). We found that differences in sample size, study method, quality grade of study, source of pre-pregnancy BMI or infant BW had a strong impact on the association between pre-pregnancy obesity and LGA, and that the factors may explain (at least in part) the heterogeneity between studies. Further studies should consider these factors and avoid such sources of heterogeneity.

Simultaneously, we assessed the quality of included studies, and found that 86.7% of the studies were of low/medium and not high quality. Therefore, an adequately powered, high-quality cohort study is needed to investigate the impact of pre-pregnancy BMI on infant BW and offspring overweight/obesity.

Finally, other factors may also have contributed to the impact of pre-pregnancy BMI on BW and offspring overweight/obesity. These factors may have been maternal age, ethnicity, gestational hypertension, gestational DM, smoking during pregnancy, educational level, and gestational weight gain (GWG) [83]–[85]. Therefore, further studies should adjust for these factors and analyze them at different levels.

The “fetal origins” hypothesis proposes that alterations in fetal nutrition results in developmental adaptations that permanently change the structure, physiology, and metabolism, thereby predisposing individuals to overweight/obesity in adulthood [86]. The process whereby a stimulus or insult at a sensitive or critical period of development has long-term effects is termed “programming” [87]. Malnutrition or over-nutrition in the mother have direct effects on the body size of the offspring, and may contribute to the risk of overweight/obesity in later life. Some studies have found that malnutrition or over-nutrition in the mother can cause epigenetic changes in humans that persist throughout life, which might explain the conclusions of our review.

The results of this review could aid better understanding of the impact of pre-pregnancy BMI on BW and offspring overweight/obesity. They could also be useful for the regulation of pre-pregnancy BMI so as to reduce the risk of overweight/obesity in offspring. A systemic review encompassing 75 articles on anti-obesity surgery showed that the risk of macrosomia could be lowered after maternal weight loss induced by surgery [88]. The pre-conception of weight loss also could reduce the risk of offspring obesity at age 7 years (OR, 0.41; 95% CI, 0.20–0.83; *P*<0.01) [89]. For underweight mothers, adequate weight gain during pregnancy could reduce the risk of LBW and SGA [90]. Hence, it might be possible to prevent the outcomes of offspring overweight/obesity by weight-regulation interventions in pre-pregnancy and pregnancy.

According to the studies included in this review, there remain some unresolved issues. Underweight mothers have a higher the risk of SGA and LBW than normal-weight mothers, and some studies have shown that infants with SGA carry an increased risk of overweight/obesity. Results from the famine in the Netherlands showed that maternal malnutrition during early gestation was associated with a higher risk of offspring overweight/obesity. Some animal studies support the association between pre-pregnancy underweight and subsequent overweight/obesity in offspring. Nevertheless, further high-quality, large-sample, mother–infant cohort studies are needed.

Pre-pregnancy overweight or obesity increased the risk of LGA, HBW, macrosomia, and later offspring overweight/obesity has been confirmed in the review. Some weight-regulating intervention studies have displayed the short-term maternal and neonatal outcomes, which indicating that interventions can help pregnant and postpartum women manage their weight, deceased the risk of LGA, HBW, macrosomia. Few trials have addressed the growth and development outcomes (offspring overweight/obesity) resulting from maternal weight loss. However, there are several ongoing randomized trials examining the impact of interventions on not only optimal maternal–fetal outcomes, but also offspring obesity [91], [92].

In addition, BW and offspring overweight/obesity are affected by maternal age, ethnicity, gestational hypertension and gestational DM, smoking during pregnancy, educational level, and GWG. Therefore, further studies assessing the impact of pre-pregnancy BMI on infant BW and offspring overweight/obesity should adjust for these factors and analyze them at different levels. Further, the impact of some important factors (GWG, smoking during pregnancy) on BW and offspring overweight/obesity need be assessed separately or interdependently with pre-pregnancy BMI. This understanding would help inform the evidence base for effective nutritional interventions in women before and during pregnancy.

In conclusion, our review suggests that, in comparison with mothers with a normal BMI: pre-pregnancy underweight increases the risk of SGA and LBW; pre-pregnancy overweight/obesity increases the risk of LGA, HBW, macrosomia, and subsequent offspring overweight/obesity. Recognition of this association may have important implications for primary prevention strategies for offspring overweight/obesity by targeting maternal pre-pregnancy BMI. However, this review also demonstrates other factors that may potentially mediate this association. These include maternal age, ethnicity, gestational hypertension and gestational DM, smoking during pregnancy, educational level, and GWG. These factors must be addressed in future studies. We also offer a developmental nutrition hypothesis on potential mechanisms involving epigenetic changes induced in the embryo. We can not confirm the association between pre-pregnancy underweight and offspring overweight/obesity according to the present study: further high-quality, large-sample, mother–infant cohort studies are needed.

## Supporting Information

Appendix S1 **Search strategy for CINAHL, EMBASE and MEDLINE databases.**
(DOC)

Appendix S2 **Quality-assessment extraction form.**
(DOC)

Appendix S3 **Quality assessment (grade) of the 45 studies included in the analysis.**
(DOC)

Appendix S4 **Funnel plot and Egger's test for a meta-analysis investigating the association between pre-pregnancy obesity and being LGA.**
(DOC)

Checklist S1 **PRISMA Checklist for the meta-analysis.**
(DOC)

Figure S1 **Forest plot of the association between pre-pregnancy BMI and LBW.**
(TIF)

Figure S2 **Forest plot of the association between pre-pregnancy BMI and HBW.**
(TIF)

Figure S3 **Forest plot of the association between pre-pregnancy BMI and macrosomia.**
(TIF)

Figure S4 **Forest plot of the association between pre-pregnancy BMI and offspring overweight and obesity.**
(TIF)

Table S1 **Characteristics of studies examining the relationship between pre-pregnancy BMI and BW.**
(DOC)

Table S2 **Characteristics of studies examining the relationship between pre-pregnancy BMI and offspring overweight/obesity.**
(DOC)
