# Supplementary material for: Pre-Pregnancy Body Mass Index in Relation to Infant Birth Weight and Offspring Overweight/Obesity: A Systematic Review and Meta-Analysis
Source: PLoS One. 2013 Apr 16;8(4):e61627. doi: 10.1371/journal.pone.0061627 (PMC3628788; doi:10.1371/journal.pone.0061627)
Supplement: Appendix S1 — Search strategy for CINAHL, EMBASE and MEDLINE databases. (DOC) [file pone.0061627.s001.doc]

**Appendix S1**

Search strategy used for CINAHL, EMBASE and MEDLINE

CINAHL database was searched using the OVID interface (http://www.ovid.com). EMBASE and MEDLINE were searched using the EMBASE interface (http://www.embase.com). The same search strategy was used by CINAHL, EMBASE and MEDLINE.

Search strategy for CINAHL, EMBASE and MEDLINE

1. exp mothers/

2. exp pregnancy/

3. exp pre-pregnancy/

4. exp gestational/

5. exp maternal /

6. or/1-5

7. obes$.af.

8. weight gain.af.

9. (overweight or over weight).af.

10. (bmi or body mass index).af.

11. or/7-10

12. 6 and 11

13. exp birth weight/

14. exp birth size/

15. exp Ponderal index/

16. (birth weight or BW).af.

17. or/12-15

18. 12 and 17

19. child$.af.

20. infant$.af.

21. exp toddler/

20. adolescenc$.af.

20. adult$.af.

21. exp offspring/

22. or/19-21

23. 11 and 22

24. 18 OR 23

25. Animals/

26. 24 not 25

27. limit 26 to yr=1970-2012
