# Supplementary material for: Pre-Pregnancy Body Mass Index in Relation to Infant Birth Weight and Offspring Overweight/Obesity: A Systematic Review and Meta-Analysis
Source: PLoS One. 2013 Apr 16;8(4):e61627. doi: 10.1371/journal.pone.0061627 (PMC3628788; doi:10.1371/journal.pone.0061627)
Supplement: Appendix S2 — Quality-assessment extraction form. (DOC) [file pone.0061627.s002.doc]

**Appendix S2**

Quality assessment extraction form

| ***1. Design of study*** | *Score* |
| --- | --- |
| Prospective | 2 |
| Retrospective | 1 |
| No information | 0 |
| **2. Loss to follow-up/completeness of records** |  |
| ≤20% | 2 |
| >20% | 1 |
| No information | 0 |
| **3. Sample size** |  |
| ≥5,000 | 2 |
| <5,000, ≥2,000  <2,000 | 1  0 |
| **4. Participant selection** |  |
| Cohort studies |  |
| Selected cohort was representative of the general population | 2 |
| Cohort was a selected group | 1 |
| Selection of the cohort group was not described | 0 |
| Case–control studies |  |
| Cases and controls were drawn from the same population | 2 |
| Cases and controls were drawn from different sources  Selection was not described | 1  0 |
| **5. Comparability of groups** |  |
| Baseline characteristics (potential confounders) |  |
| Cohort studies |  |
| No differences between the groups reported explicitly (age, parity, etc) | 2 |
| Differences between the groups reported but adjusted for | 1 |
| No information on differences between the groups | 0 |
| Differences between the groups existed, no adjustment was made | 0 |

| ***5. Comparability of groups*** | *Score* |
| --- | --- |
| Baseline characteristics (potential confounders) |  |
| Case–control studies |  |
| Controls selected from the same population as cases and independently of exposure | 2 |
| Method for selection of controls unclear or not reported | 0 |
| Controls selected from different population as cases and/or not independently of exposure | 0 |
| **6. Statistical methods** |  |
| Cohort study |  |
| Explain how loss to follow-up was addressed | 2 |
| No information on loss to follow-up | 0 |
| Case-control study |  |
| Explain how matching of cases and controls was addressed | 2 |
| No information on matching of cases and controls | 0 |
| **7. Exposure (pre-pregnancy overweight/obesity)** |  |
| Assessment of pre-pregnancy overweight/obesity |  |
| Referenced or standard definition of assessment method | 2 |
| No information on assessment method | 0 |
| **8. Outcome (birth weight/offspring overweight/obesity)** |  |
| a. Measurement of birth weight/offspring overweight or obesity |  |
| Referenced or explicit definition | 2 |
| No information or unclear on criteria used for measurement of birth weight/offspring overweight or obesity | 0 |
| b. Data extraction of birth weight/offspring overweight or obesity |  |
| Give sufficient dichotomous data for birth weight/offspring overweight or obesity | 2 |
| Unable to obtain sufficient dichotomous data on birth weight/offspring overweight or obesity | 0 |

Score: high > 14; medium = 11–14; low = <11 (maximum, 18)
