# Supplementary material for: Pre-Pregnancy Body Mass Index in Relation to Infant Birth Weight and Offspring Overweight/Obesity: A Systematic Review and Meta-Analysis
Source: PLoS One. 2013 Apr 16;8(4):e61627. doi: 10.1371/journal.pone.0061627 (PMC3628788; doi:10.1371/journal.pone.0061627)
Supplement: Appendix S3 — Quality assessment (grade) of the 45 studies included in the analysis. (DOC) [file pone.0061627.s003.doc]

**Appendix S3** Quality assessment (grade) of the 45 studies included in the meta-analysis

| *Identification* | *Final Assessment* | *Score* | *1* | *2* | *3* | *4* | *5* | *6* | *7* | *8a* | *8b* |
| --- | --- | --- | --- | --- | --- | --- | --- | --- | --- | --- | --- |
|  |  |  | Design | Loss to Follow up | Sample Size | Participant Selection | Comparability of groups | Statistical methods | Exposure | Outcome |  |
|  |  |  |  |  |  |  |  |  |  | Measurement | Data extraction |
|  | <11 Low |  | Prospective (2) | ≤20% (2) | ≥5000  (2) | Representative (2) | No differences (2) | Explain (2) | Referenced or standard definition (2) | Referenced or explicit definition (2) | Give sufficient data (2) |
|  | 11–14 Medium |  | Retrospective (1) | >20% (0) | <5000,  ≥ 2000  (1) | Selected group (1) | Differences adjusted (1) | No information (0) | No information (0) | No information (0) | Unable to obtain sufficient data (0) |
|  | >14 High |  | No information (0) | No information (0) | <2000  (0) | No information (0) | No information (0) |  |  |  |  |
|  |  |  |  |  |  |  | Differences not adjusted (0) |  |  |  |  |
| Sebire *et al*. (2001) (40) | 15 |  | 2 | 2 | 2 | 2 | 1 | 2 | 2 | 0 | 2 |
| Baeten *et al*. (2001) (41) | 13 |  | 2 | 0 | 2 | 2 | 1 | 2 | 2 | 0 | 2 |
| Jensen *et al*. (2003) (42) | 9 |  | 1 | 0 | 1 | 0 | 1 | 2 | 2 | 0 | 2 |
| Rode *et al*. (2005) (43) | 15 |  | 2 | 2 | 2 | 2 | 1 | 2 | 2 | 0 | 2 |
| Hedderson *et al*. (2006) (44) | 8 |  | 1 | 0 | 0 | 0 | 1 | 2 | 2 | 0 | 2 |
| Bhattacharya *et al*. (2007) (45) | 14 |  | 1 | 2 | 2 | 2 | 1 | 2 | 2 | 0 | 2 |
| Frederick *et al*. (2008) (46) | 10 |  | 2 | 0 | 1 | 0 | 1 | 2 | 2 | 0 | 2 |
| Leung *et al*. (2008) (47) | 12 |  | 1 | 0 | 2 | 2 | 1 | 2 | 2 | 0 | 2 |
| Gilboa *et al*. (2008) (48) | 9 |  | 1 | 0 | 1 | 0 | 1 | 2 | 2 | 0 | 2 |
| Kalk *et al*. (2009) (16) | 11 |  | 1 | 2 | 1 | 0 | 1 | 2 | 2 | 0 | 2 |
| Joy *et al*. (2009) (49) | 11 |  | 1 | 2 | 2 | 0 | 0 | 2 | 2 | 0 | 2 |
| Dietz *et al.* (2009) (50) | 15 |  | 2 | 2 | 2 | 2 | 1 | 2 | 2 | 0 | 2 |
| Oken *et al.* (2009) (51) | 11 |  | 1 | 2 | 1 | 0 | 1 | 2 | 2 | 0 | 2 |
| Meher Un *et al.* (2009) (52) | 7 |  | 2 | 0 | 0 | 0 | 1 | 0 | 2 | 0 | 2 |
| Khashan *et al.* (2009) (53) | 15 |  | 2 | 2 | 2 | 2 | 1 | 2 | 2 | 0 | 2 |
| Margerison Zilko *et al*. (2010) (54) | 12 |  | 2 | 0 | 1 | 2 | 1 | 2 | 2 | 0 | 2 |
| Aydin *et al*. (2010) (55) | 13 |  | 1 | 2 | 2 | 2 | 0 | 2 | 2 | 0 | 2 |
| Narchi *et al*. (2010) (56) | 9 |  | 2 | 0 | 2 | 0 | 1 | 0 | 2 | 0 | 2 |
| Athukorala *et al*. (2010) (57) | 10 |  | 2 | 2 | 0 | 0 | 0 | 2 | 2 | 0 | 2 |
| Mantakas *et al*. (2010) (58) | 10 |  | 1 | 0 | 2 | 0 | 1 | 2 | 2 | 0 | 2 |
| Chen *et al*. (2010) (59) | 11 |  | 1 | 2 | 1 | 0 | 1 | 2 | 2 | 0 | 2 |
| Tabatabaei *et al.* (2011) (60) | 14 |  | 1 | 2 | 2 | 2 | 1 | 2 | 2 | 0 | 2 |
| Park *et al*. (2011) (61) | 14 |  | 1 | 2 | 2 | 2 | 1 | 2 | 2 | 0 | 2 |
| Rayis *et al*. (2011) (62) | 6 |  | 1 | 0 | 0 | 0 | 1 | 0 | 2 | 0 | 2 |
| Han *et al*. (2011) (63) | 9 |  | 1 | 1 | 0 | 0 | 1 | 2 | 2 | 0 | 2 |
| Liu *et al*. (2011) (13) | 8 |  | 1 | 0 | 2 | 0 | 1 | 0 | 2 | 0 | 2 |
| Joshi *et al*. (2011) (64) | 6 |  | 1 | 0 | 0 | 0 | 1 | 0 | 2 | 0 | 2 |
| Saereeporncharenkul *et al*. (2011) (65) | 9 |  | 1 | 0 | 1 | 0 | 1 | 2 | 2 | 0 | 2 |
| Hunt *et al*. (2012) (66) | 12 |  | 1 | 0 | 2 | 2 | 1 | 2 | 2 | 0 | 2 |
| Di Benedetto *et al*. (2012) (67) | 11 |  | 1 | 2 | 1 | 0 | 1 | 2 | 2 | 0 | 2 |
| Munim *et al*. (2012) (68) | 7 |  | 1 | 0 | 1 | 0 | 1 | 0 | 2 | 0 | 2 |
| Heude *et al*. (2012) (69) | 11 |  | 1 | 2 | 1 | 0 | 1 | 2 | 2 | 0 | 2 |
| Ferraro *et al*. (2012) (70) | 12 |  | 2 | 0 | 1 | 2 | 1 | 2 | 2 | 0 | 2 |
| Jeric *et al*. (2012) (71) | 7 |  | 1 | 0 | 1 | 0 | 1 | 0 | 2 | 0 | 2 |
| Whitaker *et al.* (2004) (72) | 12 |  | 1 | 0 | 2 | 2 | 1 | 2 | 2 | 0 | 2 |
| Padez *et al.* (2005) (73) | 9 |  | 1 | 0 | 1 | 0 | 1 | 2 | 2 | 0 | 2 |
| Li *et al.* (2004) (74) | 11 |  | 1 | 0 | 1 | 2 | 1 | 2 | 2 | 0 | 2 |
| Salsberry *et al.* (2005) (75) | 11 |  | 1 | 0 | 1 | 2 | 1 | 2 | 2 | 0 | 2 |
| Dubois *et al.* (2006) (76) | 9 |  | 2 | 0 | 0 | 0 | 1 | 2 | 2 | 0 | 2 |
| Hernandez-Valero *et al.* (2007) (77) | 8 |  | 1 | 0 | 0 | 0 | 1 | 2 | 2 | 0 | 2 |
| Hawkins *et al.* (2009) (78) | 15 |  | 2 | 2 | 2 | 2 | 1 | 2 | 2 | 0 | 2 |
| Gewa *et al.* (2010) (79) | 10 |  | 1 | 0 | 0 | 2 | 1 | 2 | 2 | 0 | 2 |
| Maddah *et al.* (2010) (80) | 15 |  | 2 | 2 | 2 | 2 | 1 | 2 | 2 | 0 | 2 |
| Laitinen *et al.* (2012) (81) | 13 |  | 2 | 0 | 2 | 2 | 1 | 2 | 2 | 0 | 2 |
| Janjua *et al.* (2012) (82) | 6 |  | 1 | 0 | 0 | 0 | 1 | 0 | 2 | 0 | 2 |
