# Supplementary material for: Pre-Pregnancy Body Mass Index in Relation to Infant Birth Weight and Offspring Overweight/Obesity: A Systematic Review and Meta-Analysis
Source: PLoS One. 2013 Apr 16;8(4):e61627. doi: 10.1371/journal.pone.0061627 (PMC3628788; doi:10.1371/journal.pone.0061627)
Supplement: Appendix S4 — Funnel plot and Egger's test for a meta-analysis investigating the association between pre-pregnancy obesity and being LGA. (DOC) [file pone.0061627.s004.doc]

**Appendix S4**

Funnel plot and Egger’s test for a meta-analysis investigating the association between pre-pregnancy obesity and being LGA.

Tests for publication bias

Begg's test

adjusted Kendall's score (P-Q) = –24

Standard deviation of score = 30.82

Number of studies = 20

z = –0.78

Pr > |z| = 0.436

z = 0.75 (continuity corrected)

Pr > |z| = 0.456 (continuity corrected)

Egger’s test

----------------------------------------------------------------------------------------------------------------------

Std_Eff | Coef. Std. Err. t P>|t| [95% Conf. Interval]

-------------+--------------------------------------------------------------------------------------------------------

slope | .41712 .0278612 14.97 0.000 .3585857 .4756542

bias | .2073855 .8639817 0.24 0.813 –1.607773 2.022544

----------------------------------------------------------------------------------------------------------------------
