# Supplementary figures and images for: Pre-Pregnancy Body Mass Index in Relation to Infant Birth Weight and Offspring Overweight/Obesity: A Systematic Review and Meta-Analysis
Source: PLoS One. 2013 Apr 16;8(4):e61627. doi: 10.1371/journal.pone.0061627 (PMC3628788; doi:10.1371/journal.pone.0061627)

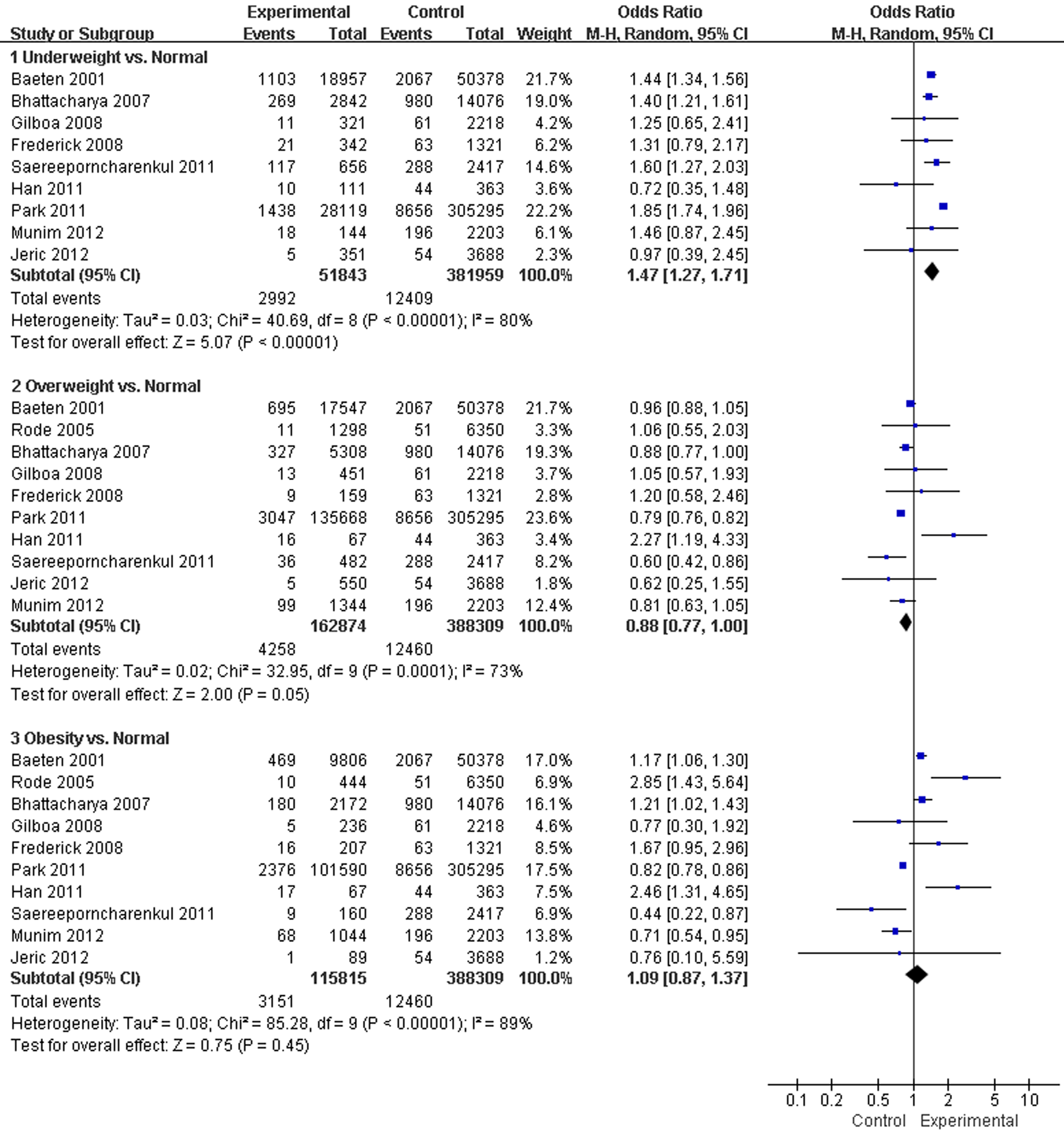

Supplement: Figure S1 — Forest plot of the association between pre-pregnancy BMI and LBW. (TIF) [file pone.0061627.s006.tif]

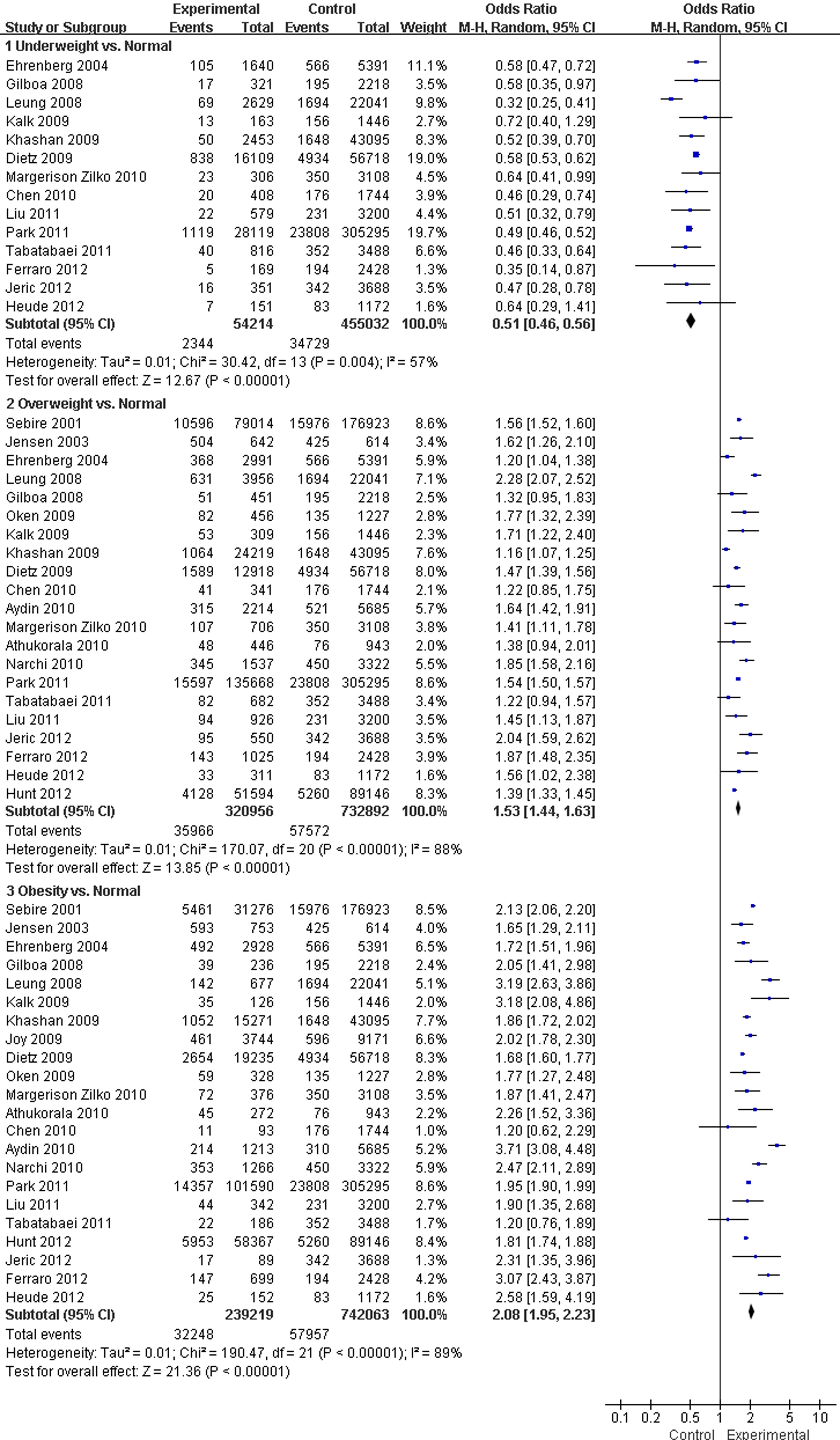

Supplement: Figure S2 — Forest plot of the association between pre-pregnancy BMI and HBW. (TIF) [file pone.0061627.s007.tif]

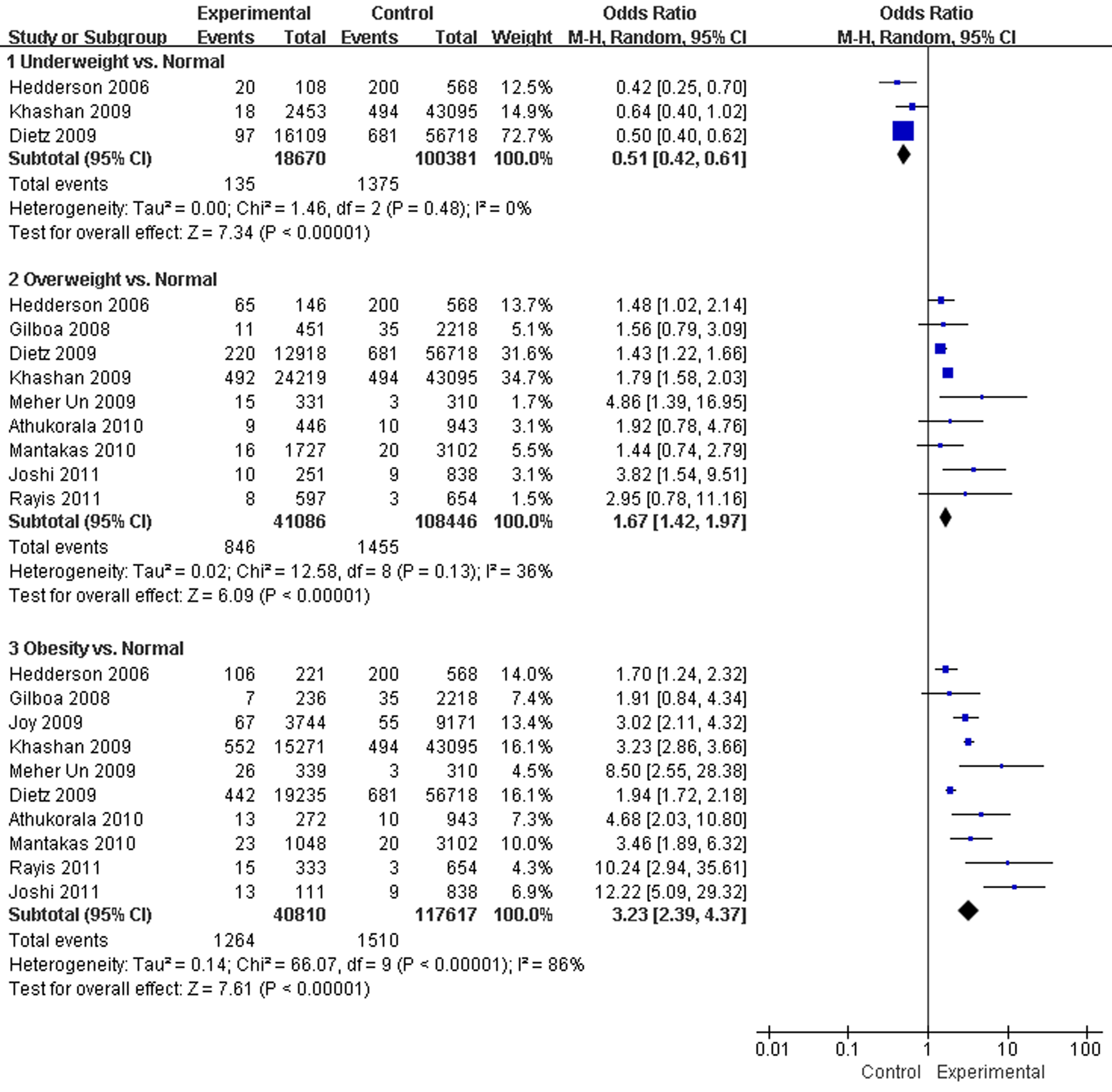

Supplement: Figure S3 — Forest plot of the association between pre-pregnancy BMI and macrosomia. (TIF) [file pone.0061627.s008.tif]

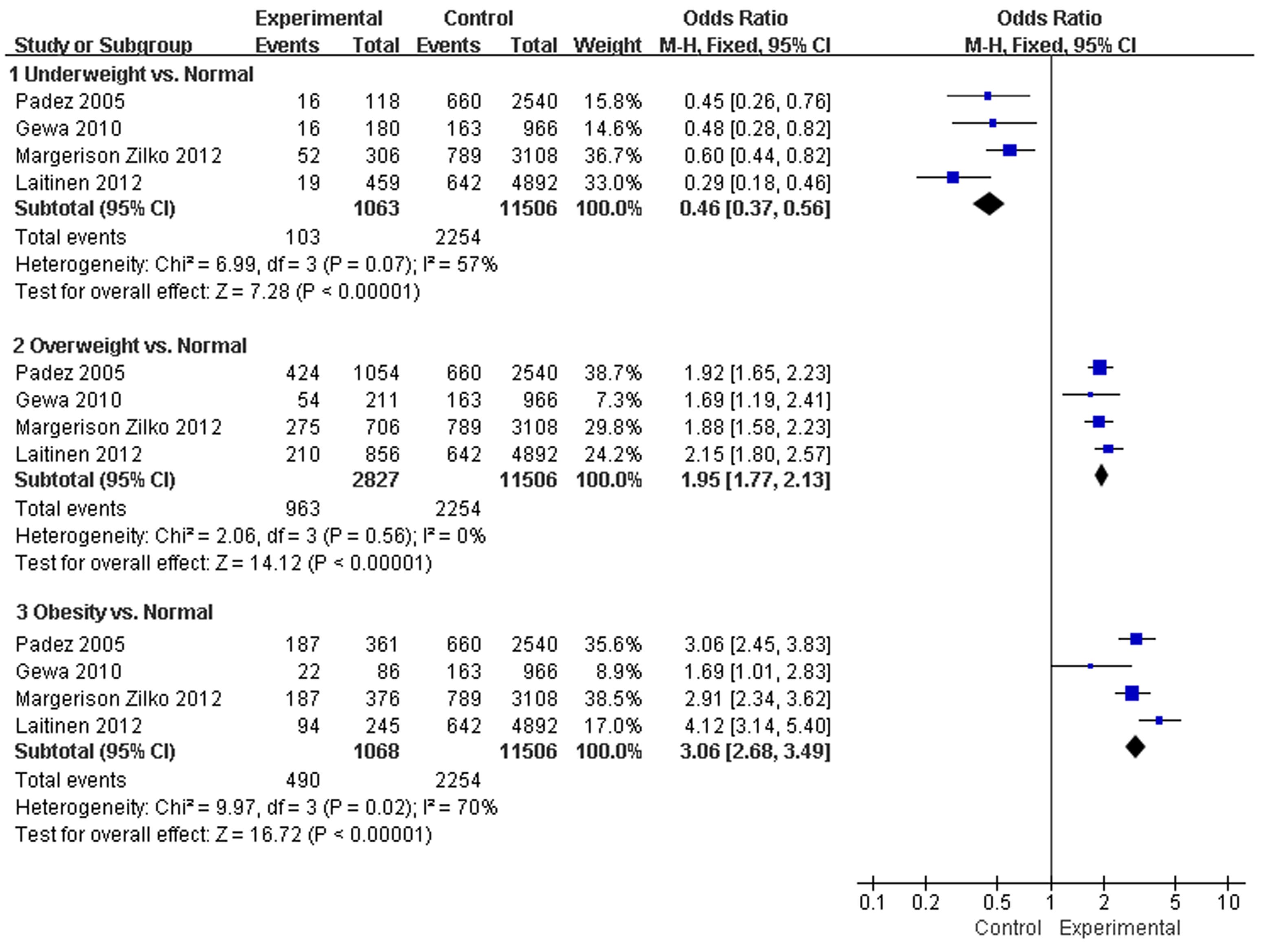

Supplement: Figure S4 — Forest plot of the association between pre-pregnancy BMI and offspring overweight and obesity. (TIF) [file pone.0061627.s009.tif]
