# Supplementary material for: Pre-Pregnancy Body Mass Index in Relation to Infant Birth Weight and Offspring Overweight/Obesity: A Systematic Review and Meta-Analysis
Source: PLoS One. 2013 Apr 16;8(4):e61627. doi: 10.1371/journal.pone.0061627 (PMC3628788; doi:10.1371/journal.pone.0061627)
Supplement: Table S1 — Characteristics of studies examining the relationship between pre-pregnancy BMI and BW. (DOC) [file pone.0061627.s010.doc]

**Table S1** Characteristics of studies examining the relationship between pre-pregnancy BMI and BW

| *Source* | *Country study period* | *Study design/source of population (n)* | *Source of BMI* | *BMI categories* | *Source of birth weight* | *Categories of birth weight* | *Confounding factors considered* |
| --- | --- | --- | --- | --- | --- | --- | --- |
| Sebire *et al*. (2001) (40) | UK 1989–1997 | Prospective cohort from St Mary’s Maternity Information System database (SMMIS) (n=287231) | Recorded from medical records | Abrams and Parker | NR | LGA | Maternal age, ethnicity,  history of hypertension and DM |
| Baeten *et al*. (2001) (41) | Sweden 1992–1997 | Prospective cohort from Washington State birth certificate tapes (n=96801) | Self-reported | Abrams and Parker | Recorded from medical records | LBW; HBW; SGA | Maternal age, smoking during pregnancy, educational level, and GWG |
| Jensen *et al*. (2003) (42) | Denmark | Retrospective cohort from 4 University Hospitals (n=2459) | Recorded from medical records | WHO | Recorded from medical records | HBW; LGA | Maternal age, GWG,  gestational age, smoking during pregnancy |
| Rode *et al*. (2005) (43) | Denmark 1998–2001 | Prospective cohort from the Copenhagen First Trimester Study (n=8092) | Self-reported | Abrams and Parker | NR | LBW; HBW | Maternal age, smoking during pregnancy, ethnicity, and type of conception |
| Hedderson *et al*. (2006) (44) | USA 1/1996–6/1998 | Retrospective case-control study from the Kaiser Permanente Medical Care Program (n=1,043) | Recorded from medical records | IOM | Recorded from medical records | Macrosomia | Race/ethnicity, maternal age |
| Bhattacharya *et al*. (2007) (45) | UK 1976–2005 | Retrospective cohort from Aberdeen Maternity and Neonatal Databank (AMND) (n=24241) | Recorded from medical records | Abrams and Parker | NR | LBW; HBW | Sociodemographic characteristics, induced labour, preterm delivery and caesarean section |
| Frederick *et al*. (2008) (46) | USA 12/1996–10/2004 | Prospective cohort study of the  risk factors of preeclampsia and gestational diabetes  mellitus  (n=2029) | Questionnaire | IOM | Recorded from medical records | LBW; HBW | Maternal age, race/ethnicity, education,  marital status, smoking status, GWG, infant sex, preterm birth, GDM, and  preeclampsia |
| Leung *et al*. (2008) (47) | China 1995–2005 | Retrospective cohort from the Obstetric Specialty Clinical Information  System (OBSCIS) (n=29303) | Questionnaire | WHO | Questionnaire | SGA; LGA | Maternal age, parity, gestational age, caesarean section, DM |
| Gilboa *et al*. (2008) (48) | USA 1981–1989 | Retrospective case-control study from the Baltimore-Washington Infant Study (BWIS) (n=3226) | Self-reported | WHO | NR | LBW; SGA; LGA; Macrosomia | Maternal age, maternal education, sex, infant race, smoking during pregnancy |
| Kalk *et al*. (2009) (16) | Germany 1/2000–12/2003 | Retrospective cohort from 1 University Hospital (n=2044) | Recorded from medical records | WHO | Recorded from medical records | SGA; LGA | Maternal age, smoking during pregnancy, child sex and gestational age |
| Joy *et al*. (2009) (49) | USA year not given | Retrospective cohort from a large perinatal database (n=12,915) | Recorded from medical records | Abrams and Parker | Recorded from medical records | HBW; LGA; Macrosomia | NR |
| Dietz *et al.* (2009) (50) | USA 2000–2005 | Prospective population-based cohort from the Pregnancy Risk Assessment Monitoring System (PRAMS) (n=104980) | Recorded from medical records | IOM | Recorded from medical records | LGA; Macrosomia | Infant sex and gestational age and maternal race/ethnicity, maternal age, education, and smoking during pregnancy |
| Oken *et al.* (2009) (51) | USA 1999–2002 | Retrospective cohort from the Project Viva (n=2011) | Self-reported | WHO | Recorded from medical records | SGA; LGA | GWG |
| Meher Un *et al.* (2009) (52) | Saudi Arabia 11/2008–6/2009 | Prospective cohort study from a maternity and children’s Hospital (n=1000) | Measured by the  on duty midwives | WHO | NR | Macrosomia | Maternal age, parity, chronic hypertension, social class |
| Khashan *et al.* (2009) (53) | UK 1/2004–12/2006 | Prospective population register-based cohort from the North Western Perinatal survey (NWPS) (n=85038) | Measurements obtained during  the first antenatal visit | WHO | NR | SGA; LGA; Macrosomia | Infant sex, maternal age, social deprivation score and ethnicity |
| Margerison Zilko *et al*. (2010) (54) | USA 1979–2006 | Prospective cohort from the National Longitudinal Survey of Youth 1979 (NLSY79)  (n=4496) | Self-reported | WHO | Reported by mothers | SGA; LGA | Race, poverty status, maternal educational attainment, maternal age, smoking during pregnancy |
| Aydin *et al*. (2010) (55) | Turkey 1/2000–12/2005 | Retrospective cohort from a obstetric clinics  (n=9,112) | Recorded from medical records | Abrams and Parker | NR | SGA; LGA | NR |
| Narchi *et al*. (2010) (56) | UK 1/2006–12/2007 | Prospective cohort from a hospital clinic (n=6,123) | Recorded from medical records | WHO | NR | SGA; LGA | Maternal age, GDM, hypertensive disorders, mode of delivery |
| Athukorala *et al*. (2010) (57) | South Australia 12/2001–1/2005 | Prospective case-control from Australian Collaborative  Trial of Supplements with antioxidants Vitamin C  and Vitamin E (n=1,672) | Hospital data  from their first antenatal visit | WHO | NR | SGA; LGA; Macrosomia | NR |
| Mantakas *et al*. (2010) (58) | UK 1/2001–11/2008 | Retrospective cohort from the local hospital database  (n=6974) | Recorded from medical records | Abrams and Parker | NR | HBW; Macrosomia | Gestational age |
| Chen *et al*. (2010) (59) | China 1/2009–6/2009 | Retrospective cohort from 2 hospital clinics  (n=2,532) | Self-reported | WGOC | NR | LGA | Mothers with DM |
| Tabatabaei *et al.* (2011) (60) | Iran 1/2007–1/2010 | Retrospective cohort from 1 urban prenatal care center (n=5,172) | Self-reported | IOM | Recorded from medical records | LGA | Mothers with DM |
| Park *et al*. (2011) (61) | USA 3/2004–12/2007 | Retrospective population-based cohort study using de-identified Florida birth certificate data (n=570,672) | Recorded from medical records | IOM | Birth certificate data | LBW; HBW; SGA; LGA | Maternal age, race/ethnicity, gestational age, education attainment, smoking status  during pregnancy |
| Rayis *et al*. (2011) (62) | Sudan 2/2008–4/2008 | Retrospective cross-sectional study from 1 hospital clinic (n=1,584) | Questionnaire | Abrams and Parker | Questionnaire | Macrosomia | Maternal age, education |
| Han *et al*. (2011) (63) | Korea 4/2006–4/2009 | Retrospective cross-sectional study from 2 hospital clinics (n= 608) | Questionnaire | APS | Questionnaire | LBW; HBW | Maternal age |
| Liu *et al*. (2011) (13) | China 2007–2009 | Retrospective population-based  cohort study from 1 hospital clinic  (n= 5,047) | Recorded from medical records | WGOC | Recorded from medical records | SGA; LGA | Maternal age, maternal education, GWG |
| Joshi *et al*. (2011) (64) | India 8/2005–9/2007 | Retrospective cohort from 2 hospital clinics  (n= 1,200) | Questionnaire | Abrams and Parker | Questionnaire | Macrosomia | Maternal age |
| Saereeporncharenkul *et al*. (2011) (65) | Thailand 1/2009–12/2009 | Retrospective cohort from 1 hospital clinic  (n= 3,715) | Recorded from medical records | WHO | NR | LBW; HBW | Maternal age |
| Hunt *et al*. (2012) (66) | USA 2007–2008 | Retrospective cohort from birth certificate data (n= 199,107) | Recorded from medical records | IOM | Birth certificate data | SGA; LGA | Maternal age, infant sex, race, smoking during pregnancy |
| Di Benedetto *et al*. (2012) (67) | Italy 1/2004–12/2009 | Retrospective cohort from 1 hospital clinic  (n=2,225) | Recorded from medical records | IOM | NR | HBW | GWG |
| Munim *et al*. (2012) (68) | Pakistan 2/2003–2/2007 | Retrospective cohort from 2 hospital clinics  (n= 4,735) | Recorded from medical records | IOM | NR | LBW | GWG |
| Heude *et al*. (2012) (69) | France 2/2003–9/2003 | Retrospective study from the Eden mother–child cohort (n= 1786) | Measured by midwife research assistants | WHO | Recorded from medical records | SGA; LGA | Maternal age and height, number of cigarettes smoked per week |
| Ferraro *et al*. (2012) (70) | Canada 10/2002–4/2009 | Prospective cohort from the Ottawa and Kingston  Birth Cohort (n= 4,321) | Questionnaire | WHO | Measured by the study nurse | SGA; LGA | GWG |
| Jeric *et al*. (2012) (71) | Croatia year not given | Retrospective cohort from 2 hospital clinics  (n= 4,678) | Recorded from medical records | WHO | Measured at birth on a scale | LBW; HBW; SGA; LGA | GWG |

NR, not reported; BMI, body mass index; BW, birth weight; DM, diabetes mellitus; WHO, World Health Organization; IOM, Institute of Medicine; WGOC, Working Group on Obesity in China; APS, Asia-Pacific standard; LGA, large for gestational age; SGA, small for gestational age; HBW, high birth weight; LBW, low birth weight; CDC, Centers for Disease Control and Prevention; GWG, gestational weight gain.
