# Supplementary material for: Pre-Pregnancy Body Mass Index in Relation to Infant Birth Weight and Offspring Overweight/Obesity: A Systematic Review and Meta-Analysis
Source: PLoS One. 2013 Apr 16;8(4):e61627. doi: 10.1371/journal.pone.0061627 (PMC3628788; doi:10.1371/journal.pone.0061627)
Supplement: Table S2 — Characteristics of studies examining the relationship between pre-pregnancy BMI and offspring overweight/obesity. (DOC) [file pone.0061627.s011.doc]

**Table S2** Characteristics of studies examining the relationship between pre-pregnancy BMI and offspring overweight/obesity

| *Source* | *Country study period* | *Study design/source of population (n)* | *Source of prepregnancy BMI* | *BMI Categories* | *Offspring age at outcomes (years)* | *Source of offspring BMI* | *Diagnostic criteria for offspring overweight/obesity* | *Confounding factors considered* |
| --- | --- | --- | --- | --- | --- | --- | --- | --- |
| Whitaker *et al.* (2004) (72) | USA 1992–1996 | Retrospective cohort from Women, Infants,  and Children  (n=8,494) | Recorded from medical records | WHO | 2, 3, 4 | Recorded from medical records | IOTF | Maternal age, sex, race/ethnicity, smoking during pregnancy, GWG, maternal education |
| Padez *et al.* (2005) (73) | Portugal 10/2002–6/2003 | Retrospective cross-sectional from children attending public schools in a districts  (n=2,043) | Questionnaire | WHO | 7-9.5 | Measured by trained technicians | IOTF | Sex, level of education, and birth weight |
| Li *et al.* (2004) (74) | US 1979–2006 | Retrospective cohort from children data of the National Longitudinal Survey of Youth  (NLSY79)  (n=2,530) | Computer-assisted personal interviewing | WHO | 2-14 | Measured by interviewers | CDC | Breast-feeding |
| Salsberry *et al.* (2005) (75) | US 1979–2002 | Retrospective cohort from children data of the National Longitudinal Survey of Youth  (NLSY79)  (n=3,022) | Computer-assisted personal interviewing | WHO | 2-7 | Observed by 3 consecutive biennial interviews | CDC | Maternal age, race/ethnicity, smoking during pregnancy, breastfeeding |
| Dubois *et al.* (2006) (76) | Canada 1998–2002 | Prospective cohort study from the Quebec  Longitudinal Study of Child Development  (QLSCD)  (n=1450) | Face-to-face interviews | WHO | 4.5 | Questionnaires | CDC | Smoking during pregnancy, monthly weight gain from birth to 5 months |
| Hernandez-Valero *et al.* (2007) (77) | US 7/2001–1/2003 | Retrospective population-based  Mexican–American cohort by  University of Texas M.D. Anderson Cancer Center  (n=438) | Structured interview | WHO | 5-18 | Structured interview | CDC | Child’s sex, age, and level of physical activity |
| Hawkins *et al.* (2009) (78) | UK 2000–2002 | Prospective nationally representative cohort study  (n=12,397) | Interviewed in the home by trained interviewers | WHO | 3 | Measured by trained interviewers | IOTF | Birth weight, child’s sex, child’s ethnicity, breastfeeding |
| Gewa *et al.* (2010) (79) | USA 4/2003–9/2003 | Retrospective data from the nationwide Demographic Health Survey (n=1,443) | Questionnaire | WHO | 3-5 | Recorded from the child health cards | W/IW | Maternal BMI, child’s size at birth, child’s stunting status and breastfeeding duration |
| Maddah *et al.* (2010) (80) | Iran 10/2006–3/2007 | Prospective Cross-sectional survey from all elementary schools in Rasht (n=6,635) | Self-administrated questionnaire | Abrams and Parker | 6-11 | Measured by interviewers | IOTF | Age, sex, maternal educational level, television viewing, mother’s employment, skipping breakfast |
| Margerison Zilko *et al.* (2010) (54) | USA 1979-2006 | Prospective cohort from the National Longitudinal Survey of Youth 1979 (NLSY79)  (n=4496) | Self-reported | WHO | 2-20 | Measured by the interviewer | CDC | Race, poverty status, maternal educational attainment, maternal age, smoking during pregnancy |
| Laitinen *et al.* (2012) (81) | Finland 7/1985–6/1986 | Prospective cohort study from two northernmost provinces of Finland (n=6,637) | Recorded from medical records | WHO | 16 | Measured by trained study personnel | IOTF | Level of hemoglobin in early pregnancy; smoking during early pregnancy; offspring sex; and level of education |
| Janjua *et al.* (2012) (82) | USA 12/1985–10/1988 | Retrospective data from a population of low-income women seeking prenatal care through the public health system in Birmingham (n=740) | Self-reported | WHO | 5 | Measured by interviewers | CDC | Child sex, gestational age, smoking during 1st trimester, maternal education, maternal age, race |

BMI, body mass index; WHO, World Health Organization; CDC, Centers for Disease Control and Prevention; IOTF, International Obesity Task Force; W/IW, ratio of weight (W) to ideal weight (IW); GWG, gestational weight gain.
